# Metabolic profiles by ^1^H-magnetic resonance spectroscopy in natalizumab-associated post-PML lesions of multiple sclerosis patients who survived progressive multifocal leukoencephalopathy (PML)

**DOI:** 10.1371/journal.pone.0176415

**Published:** 2017-04-26

**Authors:** Ruth Schneider, Barbara Bellenberg, Robert Hoepner, Eva-Maria Kolb, Gisa Ellrichmann, Aiden Haghikia, Ralf Gold, Carsten Lukas

**Affiliations:** 1 Department of Neurology of the Ruhr-University Bochum, St. Josef-Hospital, Bochum, Germany; 2 Department of Diagnostic and Interventional Radiology and Nuclear Medicine of the Ruhr-University Bochum, St. Josef-Hospital, Bochum, Germany; Julius-Maximilians-Universität Würzburg, GERMANY

## Abstract

**Purpose:**

Early diagnosis and treatment of multiple sclerosis-related progressive multifocal leukoencephalopathy (PML) significantly improve clinical outcomes. However, there is a lack of information regarding the restart of immunomodulatory therapy in the post-PML setting, when multiple sclerosis activity reappears. We aimed at the examination of metabolic differences using ^1^H-magnetic resonance spectroscopy (^1^H-MRS) in multiple sclerosis patients at various post-PML stages and at the exploration of differences according to their disease and JC virus (JCV) status.

**Methods:**

^1^H-MRS of PML lesions was carried out on 15 relapsing-remitting multiple sclerosis patients with natalizumab-associated PML. Patients were grouped according to their stage after PML infection as early post-PML, less than 19 months after PML onset (*n* = 5), or late post-PML group, more than 23 months after PML onset (*n* = 10). The latter group was further categorized according to persisting JCV load in the cerebrospinal fluid.

**Results:**

Early post-PML patients showed significantly higher Lipid/Creatine ratios within PML lesions than late post-PML (*p* = 0.036). Furthermore, N-Acetyl-Aspartate/Creatine and N-Acetyl-Aspartate/Choline were significantly reduced in early post-PML and late post-PML lesions relative to normal-appearing white matter. In late post-PML, virus-positive patients showed significantly higher ratios of Choline/Creatine (*p* = 0.019) and consequently a reduced N-Acetyl- Aspartate/Choline ratio (*p* = 0.010) in contrast to virus-negative patients. In late post-PML patients with persisting viral load, an elevated Choline/Creatine ratio correlated significantly with higher disability.

**Conclusions:**

^1^H-MRS may provide additional information related to underlying PML disease activity in various post-PML stages. In particular, Choline/Creatine levels, Lipid levels, and N-Acetyl- Aspartate/Choline are relevant markers in the post-PML setting, taking also the JCV status into account.

## Introduction

Progressive multifocal leukoencephalopathy (PML), an opportunistic infection of the central nervous system caused by the John Cuningham Virus (JCV) occurs predominantly in immune-compromised patients. Patients with Human Immunodeficiency Virus (HIV) infection are principally affected [1], but JCV also occurs as a rare side effect in natalizumab-treated patients with relapsing-remitting multiple sclerosis (RRMS) [2, 3]. Furthermore, single cases of PML associated with other disease-modifying therapies (dimethyl fumarate and fingolimod) treatment have been described [4, 5].

The clinical outcome of natalizumab-associated PML patients with reported survival rates of 76% to 80% [6] (https://www.biogenidec.com) is much better than in HIV-PML or other populations, such as transplant recipients [7], and standardized therapy regimes during the course of immune reconstitution inflammatory syndrome (IRIS) have been recommended [8]. Therapeutic interventions include plasma exchange or immunoadsorption to accelerate the elimination of the monoclonal antibody of natalizumab and dedicated treatment by corticosteroid therapy during IRIS. Additionally, based on expert recommendations only, mefloquine and mirtazapine were used, both inducing possible inhibitory effects on virus replication, although precise treatment effects for both drugs remain unclear [3, 9–11]. Longitudinal observations have shown that in PML survivors functional disability usually stabilizes at reduced levels after about six months and tends to stay stable beyond 18 months after PML diagnosis unless new MS relapses occur [6, 8]. However, there is a lack of information regarding the restart of immunomodulatory therapy in the post-PML setting, when MS activity reappears. In a recent study, a restart of immunomodulatory treatment in post-PML was recommended when new MS disease activity occurs and cerebrospinal fluid (CSF) is proven JCV-free [8].

Unlike other magnetic resonance (MR) techniques, proton MR spectroscopy (^1^H-MRS) offers the opportunity to characterize metabolic changes in pathologic lesions as well as in normal-appearing brain tissue [12]. To date, only a few case reports describe ^1^H-MRS findings in natalizumab-associated PML, bearing no relation to the post-PML setting [13–15]. In HIV-associated PML typical ^1^H-MRS findings include reduced N-Acetylasparate (NAA) and increased Choline (Cho) and Lipid (Lip) levels with especially elevated metabolite ratios of Cho and Lip to Creatine (Cr) during the IRIS phase [16, 17]. Investigating metabolic changes in post-PML lesions could be useful to assess ongoing disease activity in this phase of clinical stabilization and may provide additional information to estimate inflammatory conditions in the post-PML setting. Thus, the primary goals of this MRS study were the examination of metabolic profiles in PML lesions of MS patients in the post-PML setting, and the exploration of differences according to their JCV status.

## Material and methods

### Patients and treatment

Fifteen RRMS patients were included (9 female, 6 male) between June 2014 and September 2015, who were referred to our clinic for clinical re-evaluation at different stages after a natalizumab-associated PML. They received MR imaging and ^1^H-MRS in the post-PML setting. The details of the former PML diagnosis and disease course and the functional outcome of the study population have been reported recently [18] [8, 19]. Summarizing, after the onset of neurological symptoms consistent with PML, the diagnosis was verified by JCV DNA detection in the CSF in 14 patients, consistent with a diagnostic accuracy of “definitive” according to the PML consensus statement of the American Association of Neurology (AAN) [20]. In one CSF JCV-negative patient, the clinical presentation and MRI findings were indicative of PML (diagnostic accuracy “possible”) and no other differential diagnosis was found [20]. CSF samples after lumbar puncture were analyzed by PCR for JCV DNA at the Department of Virology, University of Düsseldorf. In all included patients IRIS, associated with gadolinium enhancement in PML lesions, was observed. After PML diagnosis all patients received plasma exchange/immunoadsorption to remove natalizumab and supportive treatment with mefloquine and mirtazapine [8].

Clinical disability during the course of disease was extracted from the patient files and measured by the Expanded Disability Status Scale (EDSS) and Karnofsky Performance Scale (KPS) [21, 22], one year before the PML diagnosis, at PML diagnosis and, post-PML, six to twelve months after the PML diagnosis, and at the time of the ^1^H-MRS examination. The residual PML-related functional worsening (KPS drop, EDSS rise) was expressed as the EDSS and KPS difference between scoring one year before PML diagnosis and scoring at the time of MR acquisition.

The study was approved by the ethics committee of the Ruhr-University Bochum (approval no. 4566–13) and was conducted according to the principles expressed in the Declaration of Helsinki. Written informed consent was obtained from all patients.

### Proton magnetic resonance spectroscopy (^1^H-MRS) and MR imaging

^1^H-MRS and MRI of the brain were performed on a single 3 Tesla MRI System (Philips Achieva, Best, The Netherlands) using a 32-channel head matrix coil. To localize the PML lesions and positioning of the volume of interest (VOI) for MRS, isotropic 3D sequences were performed, consisting of sagittal T1 FFE (TR/TE: 10/4.6ms, TI: 1000ms, ETL 164, matrix 240x240, 180 slices, resolution 1x1x1mm) with and without Gadolinium administration, and sagittal FLAIR (TR/TE: 4800/291ms, TI: 1650ms, ETL 182, matrix 240x240, 170 slices, resolution 1x1x1mm). For MRS, a 2D-PRESS chemical shift imaging sequence (TR/TE: 2000/45 ms, bandwidth 2000 Hz, 1024 spectral points, 128 measurements) covering an axial field of view (230x190x15 mm; resolution 10x10x15 mm) was used.

**Fig 1:**
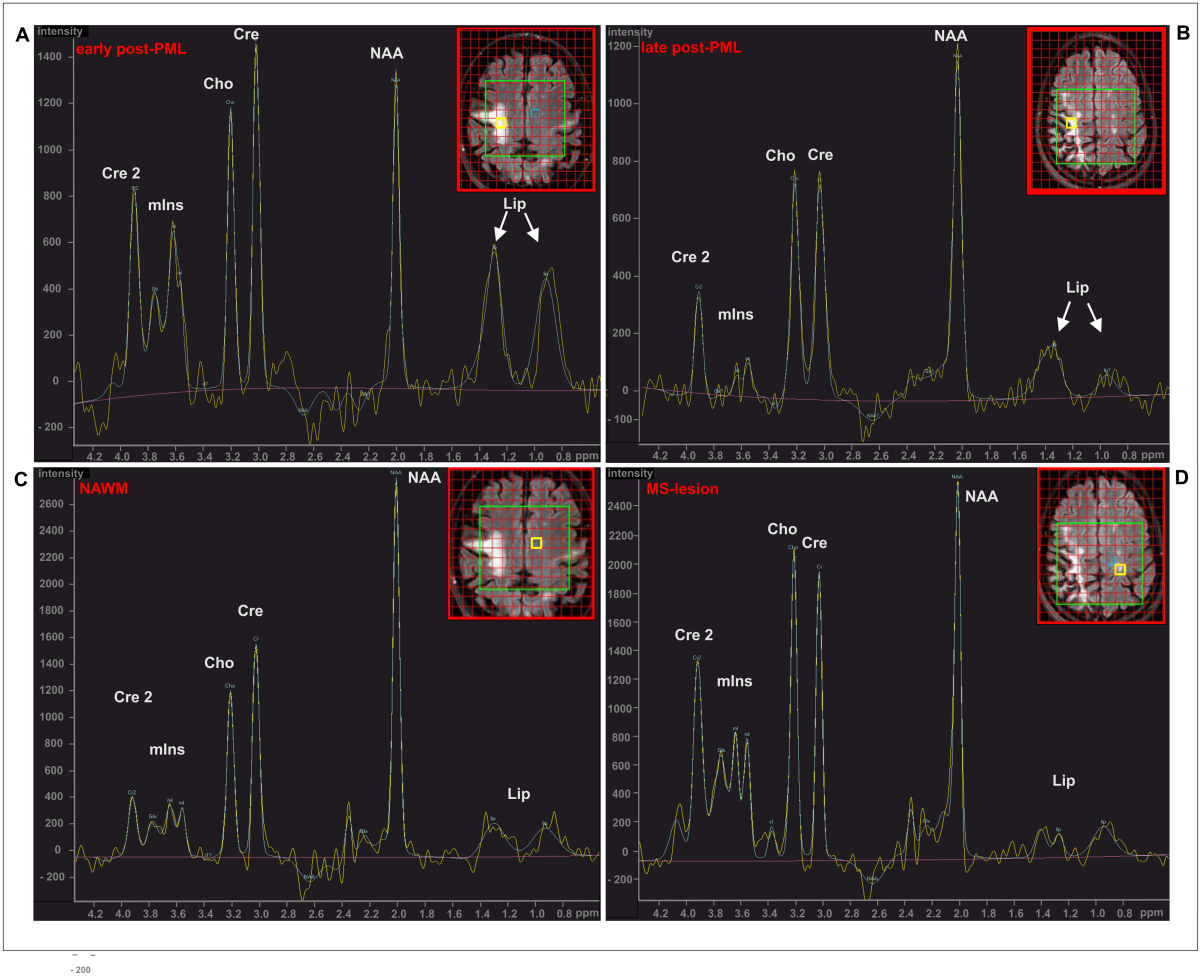
Exemplary ^1^H-spectra (2D-PRESS, TR/TE: 2000 / 45 ms). (A) PML lesion early post-PML (E-pPML, male, aged 62 years, 8 months post-PML); (B) PML lesion late post-PML (L-pPML, female, aged 38 years, 27 months post-PML); (C) NAWM (same patient as (A)); (D) MS lesion (same patient as (B)). The location of the spectra is marked by yellow squares on the inserted axial FLAIR images.

The VOI (82x90x15 mm) was positioned apart from air-tissue interfaces and surrounding bones and fat, aiming to cover the PML lesions and normal appearing white matter (NAWM) If possible, single MS lesions in white matter regions which were apparently not affected by PML were also covered. The inserted axial FLAIR images in Fig 1 illustrate typical positioning of the VOI covering PML lesions as well as NAWM. Outer volume suppression by circular saturation slices was used. The preparation phase of MRS included automatic procedures for water suppression, shimming, and tuning of the radiofrequency and gradient system and an acquisition without water suppression for correction of magnetic field distortions.

Identification of the PML lesion and differentiation from MS lesions was performed by visible comparison of pre-existing external MRI investigations before the onset of PML in each patient by an experienced rater (RS, CL). Lesion location, lesion morphology, and (if DWI images were availyble) diffusion restriction was used to differentiate PML lesions from MS lesions.

An MRI sum score was obtained at the time of MRS to semi-quantitatively describe the extension of the PML lesions depending on the affected brain regions of FLAIR-weighted imaging (range 1–9, from focal lesions affecting one gyrus to bi-hemispheric lesions covering both hemispheres).

### Spectral processing

MRS signals were post-processed using the vendor’s automatic software SpectroView (Philips, Best, The Netherlands) implemented on an external workstation [23, 24]. The automatic processing included time domain filtering (Gaussian multiplication: 5Hz and exponential multiplication: -3Hz), residual water suppression by high-pass filtering (width 30 Hz), and fast Fourier transformation. SpectroView quantifies the frequency domain data by phase correction and fitting of peaks by using prior knowledge of the expected metabolites. The baseline is fitted with a polynomial function and subtracted from the whole spectrum.

Metabolite ratios were calculated based on the peak integrals of N-Acetylaspartate (NAA, 2.01 ppm), Creatine (Cr and Cr2, 3.03 and 3.93 ppm), Choline (Cho, 3.23 ppm), and the summed peak areas of myo-Inositol (mI, 3.58 and 3.64 ppm) and Lipid (Lip, 0.9 and 1.4 ppm). For each patient, the spectral analysis included metabolite ratios extracted in the center of the PML lesions, in contralateral NAWM, and in non-PML MS lesions, if present.

### Statistical analysis

All statistical analyses were performed using SPSS 22 (IBM SPSS, Chicago, USA). Group differences between patient subgroups were explored by Mann-Whitney-U tests for EDSS, KPS, and metabolite ratios. Group differences regarding age and disease duration were assessed using Student’s t-test. Differences between the numbers of patients with JCV DNA elimination and initiation of an immune-modulatory therapy at the time of MRS were explored by Pearson’s chi-square tests. Because of the explorative character of the study and small group sizes, correction for multiple comparisons was omitted. Associations between MRS results and clinical parameters including the EDSS and KPS as well as JCV load and PML lesion extension score were conducted using Spearman correlation analyses.

## Results

### Patients: Clinical status and grouping according to JCV status and post-PML time

The patients’ demography and clinical status are summarized in Table 1; details are supplied in S1 Table. At the time of ^1^H-MRS (8 months to 67 months after PML diagnosis according to the AAN criteria) 11 patients (73%) had successfully eliminated the JCV, while in four patients (time after PML diagnosis: 40.5±15.2 months [mean±standard deviation (SD)]) the CSF examinations remained JCV-positive. To compare those patients who had a longstanding (more than 23 months) persistent JCV-positive status (late post-PML JCV+) with matched JCV-negative patients (JCV-), we classified the JCV patients according to the time interval between the PML diagnosis and the MR date (disease duration at MRS). Thus, we formed a late post-PML group (L-pPML; *n* = 10 patients; time after PML diagnosis: 38.0 [23.0–67.0] months [median, range]) and an early post-PML group (E-pPML; *n* = 5 patients; time after PML diagnosis: 12.0 [8.0–19.0] months [median, range]). Using this stratification, there were no significant differences in the distribution of the disease duration at MRS between the L-pPML and the late post-PML JCV+ groups (*p* = 0.914). In contrast, there was no significant overlap between the distributions of the E-pPML and L-pPML groups (*p* = 0.001). Therefore, this classification was also used to inspect group differences according to the disease duration at MRS examination between the early and late pPML group.

**Table 1:** Demography and clinical status of 15 RRMS patients in the post-PML phase.

		early post-PML (E-pPML)	late post-PML (L-pPML)	*p*-value	late post-PML JCV-	late post-PML JCV+	*p*-value
**PML disease duration at MRS months)**	median [range]	12 [8–19]	38 [23–67]	0.001	38 [23–67]	40 [26–56]	0.914
***n***		5	10		6	4	
**female**	no.,(%)	2 (40%)	7 (70%)		7 (70%)	2 (40%)	
**age (years)**	mean±SD	39±16	40 ±5	0.761^a^	41 ±6	38±4	0.407^a^
**MS disease duration until PML diagnosis (years)**	mean±SD	12.8±8.9	12.3±4.2	0.909^a^	11.7±4.4	11.0±4.8	0.826^a^
**EDSS change (1y before PML—time of MR)**	median [range]	-1.5 [0;-4.5]	-3.0 [-1.5;-6.5]	0.254^b^	-2.5 [0;-4.5]	-4.0 [-2;-6.5]	0.171^b^
**KPS change (1y before PML—time of MR)**	median [range]	-10 [0; -30]	-20 [-10; -60]	0.165^b^	-15 [0; -40]	-40 [-20; -60]	**0.038**^b^
**JCV elimination at time of MR**	no.(%)	5 (100%)	6 (60%)	0.099^c^	6 (100%)	0 (0%)	**0.002**^c^
**IMT post-PML at time of MR**	no.(%)	2 (40%)	6 (60%)	0.464^c^	4 (67%)	2 (50%)	0.598^c^

SD: standard deviation; IMT: immunomodulatory therapy; KPS: Karnofsky performance scale, EDSS: extended disability status scale JCV+, JCV-: JCV DNA in CSF positive/negative; RRMS: relapsing-remitting multiple sclerosis;

*p*-values: significance of group differences;

^a^: t-tests;

^b^: Mann-Whitney-U-tests;

^c^: Pearson’s chi-square tests.

When comparing E-pMPL and L-pPML patients (Table 1), there were no statistically significant differences regarding age, disease duration before the onset of PML, and change of EDSS and Karnofsky-performance scale between PML diagnosis and time of MRS (Table 1). When subdividing L-pPML patients according to their JCV status, a significantly more severe KPS drop was found in late post-PML JCV+ patients, indicating more persistent disability in this group as compared to JCV-negative post-PML patients (late post-PML JCV-, see Table 1).

In the E-pPML group, an immunomodulatory treatment (IMT) was re-initiated in two patients (40%) by using dimethyl fumarate and interferon beta-1a before the MR examination. In all JCV- patients of the L-pPML group an immunomodulatory treatment (dimethyl fumarate, fingolimod, interferon beta-1a, glatiramer acetat) was restarted before the MR examination. In two of these patients (33%) the IMT was interrupted at the time of MR examination. In two patients of the JCV+ group, IMT had not yet been initialized at the time of MRS. One was free of IMT at MR examination after a transient period of glatiramer acetat treatment, and the other patient was treated with intravenous immunoglobulin at the time of MRS (IMT initialization with interferon beta-1a, later replaced by dimethyl fumarate). There were no significant differences either between the fractions of treated and untreated patients or between the JCV+ and JCV-, or E-pPML and L-pPML groups (Table 1).

The overall survival rate of this study population was 100% [18, 19].

### ^1^H-MRS findings

Fig 1 illustrates typical MRS spectra found in PML lesions (Fig 1A: E-pPML, 8 months post PML diagnosis, and Fig 1B: L-pPML, 27 months post PML diagnosis); NAWM (Fig 1C, same patient as in A); and a MS lesion which was apparently not affected by the PML (Fig 1D, same patient as in B). It depicts elevated levels of free lipid signal in the PML lesion of the E-pPML patient and, to a lesser extent, in the L-pPML patient when compared to the NAWM spectrum in Fig 1C. The NAWM spectrum and the spectrum of the MS lesions of the PML patients depicted in Fig 1 showed typical metabolic patterns reported for healthy brain white matter (staircase-like proportion of the peak heights of Cho, Cr, and NAA) and MS lesions (relative reduction of NAA/Cr and possible increase of Cho/Cr) with similarly low lipid levels in both spectra [25] For comparison, representative MRS spectra of normal supratentorial white matter of a representative healthy control and of a white matter MS lesion of a RRMS patient without PML are provided as supplementary material (S1 Fig) [25].

Quantitative MRS analysis results are given in Table 2. Since no statistically significant differences between the post-PML subgroups were detected in the spectral results of MS lesions and NAWM, the spectral results of MS lesions (*n* = 5) and of NAWM (*n* = 14) were pooled into single groups. Comparing metabolite ratios of NAA, Cho, and Lip to Cr as well as the NAA/Cho ratio between the spectra acquired in PML lesions in the E-pPML and L-pPML group and the NAWM results, there was a significantly elevated Lip/Cr ratio in E-pPML compared to L-pPML (*p* = 0.036) and to NAWM (*p* = 0.008). Furthermore, the levels of NAA/Cr and NAA/Cho were significantly reduced in E-pPML (*p* = 0.044/ *p* = 0.044) and in L-pPML (*p* = 0.002/ *p* = 0.006) lesions relative to NAWM. Because of the marked fraction of patients who could not eliminate JCV in the CSF in the L-pPML group (40%; 4/10 patients), we compared the metabolite ratios between JCV-positive (*n* = 4) and JCV-negative (*n* = 6) patients. We observed a significantly different Cho/Cr ratio (*p* = 0.019) in the sense of higher Cho/Cr in the JCV-positive group and a consequently reduced NAA/Cho ratio in JCV-positive patients compared to JCV-negative patients (*p* = 0.010), probably reflecting different Choline levels. Furthermore, the Lip/Cr ratio was significantly increased in the MS lesions of PML patients compared to NAWM (*p* = 0.034).

**Table 2:** Spectroscopy results. Metabolite ratios in subgroups of PML lesions, MS lesions, and NAWM.

metabolite ratio median [range]	early post-PML (E-pPML) *n* = 5	late post-PML (L-pPML) *n* = 10	late post-PML JCV - *n* = 6	late post-PML JCV+ *n* = 4	all NAWM *n* = 14	all MS *n* = 5
**NAA/Cr**	1.07 [0.88,1.74] ^a^**p = 0.044** ^b^p = 0.095	1.26 [0.92,1.44] ^a^**p = 0.002** ^b^**p = 0.040** ^c^p = 0.513	1.28 [1.02,1.44] ^d^p = 0.171 ^c^p = 0.329	1.03 [0.92,1.29]	1.52 [1.21,1.88]	1.58 [1.21,2.13] ^a^p = 0.500
**Cho/Cr**	0.80 [0.66,1.26] ^a^p = 0.500 ^b^p = 0.690	0.85 [0.66,1.21] ^a^p = 0.108 ^b^p = 0.953 ^c^p = 0.440	0.74 [0.65,0.85] ^d^**p = 0.019** ^c^p = 0.247	0.99 [0.85,1.21]	0.76 [0.45,1.04]	0.81 [0.67,1.33] ^a^p = 0.219
**Lip /Cr**	1.14 [0.6,1.77] ^a^**p = 0.008** ^b^p = 0.413	0.36 [0.12,0.97] ^a^p = 0.403 ^b^p = 0.099 ^c^**p = 0.036**	0.52 [0.12,0.97] ^d^p = 0.730 ^c^p = 0.095	0.28 [0.22,0.44]	0.45 [0.28,1.06]	0.70 [0.48,1.65] ^a^**p = 0.034**
**NAA/Cho**	1.52 [0.85,2.51] ^a^**p = 0.044** ^b^p = 0.421	1.5 [0.87,1.96] ^a^**p = 0.006** ^b^p = 0.254 ^c^p = 0.954	1.73 [1.52,1.96] ^d^**p = 0.010** ^c^p = 0.177	0.99 [0.87,1.49]	2.14 [1.3,3.23]	1.73 [1.02,2.45] ^a^p = 0.391

JCV+, JCV-: JCV DNA in CSF positive/negative;

*p*-values: significance of group differences by Mann-Whitney-U tests:

^a^ = compared to NAWM;

^b^ = compared to MS lesions;

^c^ = compared to E-pPML;

^d^ = comparison JCV+ vs. JCV-

**bold**: significant with *p*<0.050

### Correlation analysis of ^1^H-MRS metabolites and EDSS/ KPS

**Fig 2:**
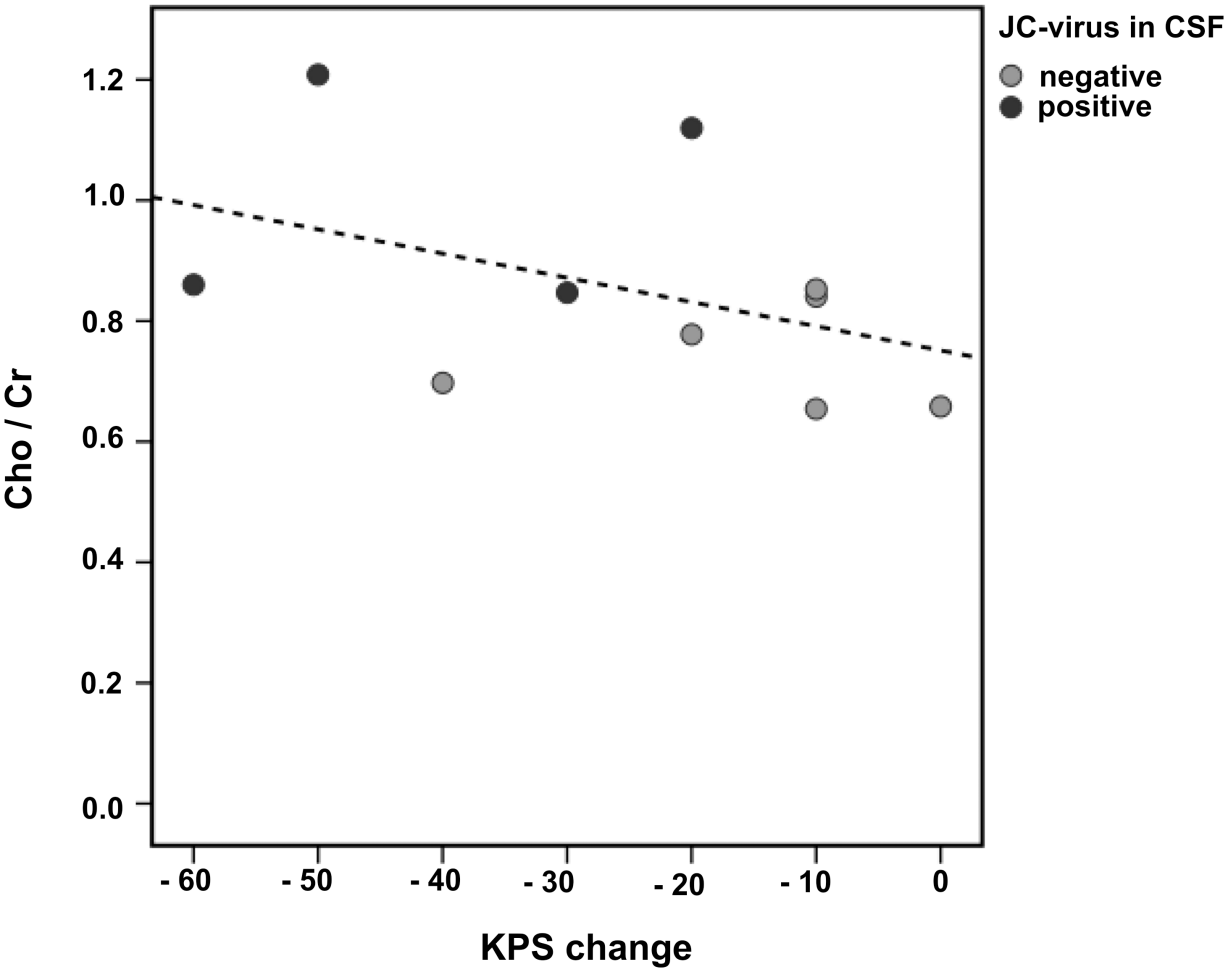
Association between Cho/Cr (in PML lesions) and the PML-related change in KPS in late post-PML patients (L-pPML). The KPS change was calculated as the difference between the time points: one year before PML diagnosis and the time of the MRS examination. The dotted line depicts a linear regression.

A significant positive correlation between the Cho/Cr ratio and KPS change was found in L-pPML patients (Fig 2, Spearman correlation *rho* = -0.566, *p* = 0.044), relating more severe residual PML-related functional worsening to higher Cho/Cr levels in the PML lesions of late post-PML patients. There was no further significant association between any ^1^H-MRS metabolite ratio and clinical parameters such as the EDSS, the KPS, the PML lesion extension score at MRS, or the JCV load at PML diagnosis in any of the patient groups.

## Discussion

Results of the current ^1^H-MRS study indicate different MRS patterns of metabolite profile changes in the post-PML setting of MS patients. The spectra of PML lesions compared to the spectra of NAWM in patients in the E-pPML phase were dominated by marked lipid peaks and reduced NAA levels, reflected by elevated Lip/Cr and low values of NAA/Cr and NAA/Cho.

By contrast, metabolic profiles in PML lesions of L-pPML patients showed normal Lip/Cr levels while low NAA/Cr and NAA/Cho persisted compared to spectra obtained in NAWM.

NAA, an amino-acid derivate exclusively synthesized in neurons, which is distributed along the anterograde axonal transport, is usually interpreted as a marker of neuronal viability, especially for axonal integrity in the white matter [12, 26], while increased Cr has been ascribed to gliosis [27]. As previously described in HIV-related PML patients and in a case report of a single MS patient with natalizumab-associated PML, the decreased NAA/Cr ratio returned to baseline during the course of PML-IRIS, suggesting that neuronal dysfunction may in part be reversible [17, 28]. Our study indicates that similar dynamic changes exist in natalizumab-associated PML patients. In the PML lesions of E-pPML as well as in L-pPML NAA/Cr was reduced, probably due to partially persistent axonal integrity loss. This was also reflected by the accordant reduced NAA/Cho ratio in both groups of post-PML patients.

The most relevant metabolite in PML lesions of post-PML patients seems to be the marked elevated lipid peak (Lip/Cr) in E-pPML patients, which returned to normal levels in L-pPML. Lipid resonances are interpreted as primary markers of cell membrane breakdown [29] and are linked to the presence of immune cells, including T cells in vitro [30, 31]. Therefore, high MRS lipid signals are interpreted as markers of activated lymphocytes [32]. In HIV-related PML lesions, increased Lip/Cr ratios were regarded as a result of the infiltration of activated T cells in the PML lesions [17]. Applying this information to post-PML MS-patients, our results suggest that elevated lipid levels may indicate an ongoing inflammatory process, which is regressive in the later post-PML phase. Correspondingly, the lipid peak might serve as a surrogate marker for persisting inflammation within PML lesions.

Another important finding of our study is related to the JCV status of PML patients in the later stage. We explored spectroscopic differences in PML lesions between patients who had eliminated the JCV and patients with persisting JCV load in the L-pPML group. Specifically, patients with persisting JCV load in the CSF showed increased Cho/Cr levels and a respectively decreased NAA/Cho ratio compared to patients with negative JCV load in the CSF. The Choline peak is determined by several Cho-containing phospholipids of cell membranes, in this regard predominantly of myelin. Regarding neuroinflammatory diseases, elevated MRS Choline levels represent abnormal Choline mobility, in turn suggesting demyelination or remyelination and inflammation [12, 33]. In PML the grade of increased Cho/Cr levels may reflect ongoing disease activity, which in terms of JCV persistence may indicate continuous inflammatory processes aiming at the complete elimination of the virus. Despite the explorative character of this study with small patient subgroups, these findings indicate that increased Cho/Cr may be related to inflammatory activity. In accordance with the hypothesis that elevated Cho/Cr ratios may represent ongoing inflammation, a significant correlation between Cho/Cr and worsening of clinical performance during the PML (KPS change between the MRS time and a year before PML diagnosis) could be shown in L-pPML, suggesting that these MRS findings are clinically relevant.

In addition, the 2D-spectroscopic technique allowed us to investigate a few focal MS lesions. In these non-PML MS lesions we found slightly elevated Lip/Cr levels compared to NAWM without other PML-like spectral changes. Given the sparse nature of MRS studies, there is a lack of comparable MRS findings in pure MS lesions of PML patients. Sinnecker et al. demonstrated moderately decreased levels of NAA in an exemplary MS lesion in a case study of one PML patient [15]. Even though free lipid signal has previously been reported in localized active MS lesions [34], in the MS lesions of patients with prior PML a widespread affection of brain white matter by inflammatory processes, extending beyond the visual location of PML lesions, has to be taken into consideration as an alternative source of lipid elevation. The major metabolite ratios (NAA/Cr, NAA/Cho, Cho/Cr) in MS lesions did not significantly differ from NAWM, which was in accordance with a recent larger MRS study in which a high similarity in the individual spectra of chronic MS lesions and contralateral NAWM was described [35]. These results, however, should be interpreted with caution, due to the low number of MS lesions studied. Given the herein used 2D MRS technique, we cannot fully exclude that data from MS white-matter lesions were ‘contaminated’ by volume averaging from adjacent NAWM, CSF, or nearby gray matter.

A few more limitations of our study have to be acknowledged. Firstly, the statistical power of the analysis was limited due to small patient subgroups in this exploratory study. Nevertheless, given that larger MRS studies in natalizumab-associated PML cases are lacking, our results are, in part, in line with previous MRS findings in HIV-related PML patients, suggesting similar metabolic profile changes in the course of PML [17].

Secondly, we compared independent groups of RRMS patients who were in early and late disease states after PML to draw conclusions about ^1^H-MRS metabolite changes over time. This exploratory cross-sectional approach seems appropriate for a pilot study; however, future longitudinal studies with larger groups of MS patients with natalizumab-associated PML are required in order to evaluate the temporal changes of metabolic patterns in the post-PML setting. Furthermore, additional information about spectroscopy findings in the course of early PML and PML-IRIS are required.

In post-PML patients, especially in non-eliminators of the JCV, the restart of immunomodulatory therapy when MS activity reappears is a difficult and precarious decision. Admittedly there is a non-evidence based recommendation to restart IMT when the CSF is proven JCV-free; nonetheless, in cases of early reappearing MS activity and occurring relapses, therapeutic intervention can become necessary [8]. Different exemplary proposals regarding post-PML MS therapy exist, which points to the fragile immunological situation in post-PML RRMS patients [36]. The present exploratory study including a relatively small number of PML patients indicated that ^1^H-MRS of PML lesions, especially regarding Cho/Cr levels in JCV-positive post-PML patients, as well as lipid levels and NAA/Cho in the post-PML period, can provide additional and potentially clinically relevant information related to ongoing PML disease activity. In the future, additional longitudinal MRS studies including higher numbers of PML patients are required to investigate the clinical relevance of MRS results in the course of the disease. Potentially, ^1^H-MRS might contribute to the evaluation of the inflammatory situation of MS patients in the post-PML setting when re-initiation the immunomodulatory therapy is being discussed.

## Supporting information

S1 Fig Examples of typical white matter ^1^H-MRS spectra (2D-PRESS, TR/TE: 2000 / 45 ms). (A) a healthy participant (male, 38 years old); and (B) MS lesion of a RRMS patient (male, 34 years old, disease duration 2 years) showing reduction of NAA/Cr and NAA/Cho and increase of Cho/Cr in MS compared to the healthy control, and low lipid levels in both spectra.
(TIF)

S1 Table Details of patients’ demography, clinical status, and MRS results. (PDF)
