# Supplementary figures and images for: Metabolic profiles by 1H-magnetic resonance spectroscopy in natalizumab-associated post-PML lesions of multiple sclerosis patients who survived progressive multifocal leukoencephalopathy (PML)
Source: PLoS One. 2017 Apr 26;12(4):e0176415. doi: 10.1371/journal.pone.0176415 (PMC5405920; doi:10.1371/journal.pone.0176415)

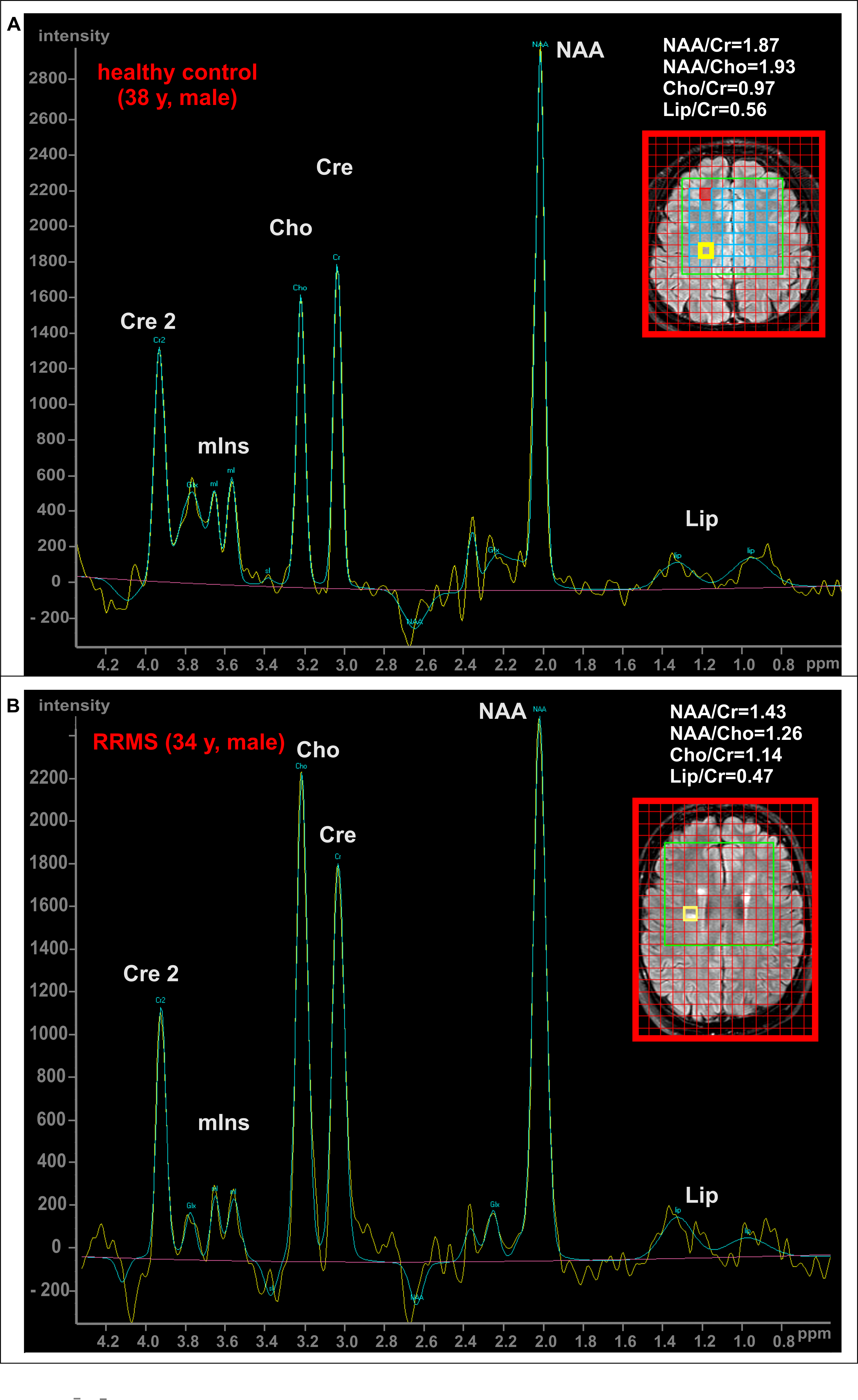

Supplement: S1 Fig — (A) a healthy participant (male, 38 years old); and (B) MS lesion of a RRMS patient (male, 34 years old, disease duration 2 years) showing reduction of NAA/Cr and NAA/Cho and increase of Cho/Cr in MS compared to the healthy control, and low lipid levels in both spectra. (TIF) [file pone.0176415.s001.tif]
