# Supplementary material for: Metabolic profiles by 1H-magnetic resonance spectroscopy in natalizumab-associated post-PML lesions of multiple sclerosis patients who survived progressive multifocal leukoencephalopathy (PML)
Source: PLoS One. 2017 Apr 26;12(4):e0176415. doi: 10.1371/journal.pone.0176415 (PMC5405920; doi:10.1371/journal.pone.0176415)
Supplement: S1 Table — (PDF) [file pone.0176415.s002.pdf]

patient nr	age at MRS (years)	male / female	MS subtype	duration between MS diagnosis and PML (years)	PML disease duration at MRS (months)	PML group	natalizumab infusions	JCV load (CSF at PML diagnosis)	JCV load (CSF at MRS)	DMT at MRS	PML lesion extension score[A] (0 - 9; on FLAIR images at time of MRS)	EDSS at MRS	EDSS at 1year before PML	EDSS at PML diagnosis	EDSS 6-12 months after PML diagnosis	KPS at MRS	KPS at 1year before PML	KPS at PML diagnosis	KPS 6-12 months after PML diagnosis	NAA/Cr			NAA/Cho			Cho/Cr			Lip /Cr		
																				PML	NAWM	MS	PML	NAWM	MS	PML	NAWM	MS	PML	NAWM	MS
1	62	m	RRMS	19	8	E-pPML	96	5		no	4	5.5	2	4	5.5	50	90	80	50	1.07	1.34		0.85	1.30		1.26	1.03		0.60	0.28	
2	43	m	RRMS	23	11	E-pPML	55	150		no	6	6.5	2	4	6	50	70	50	50	1.22	1.37		1.52	1.71		0.80	0.80		1.48	0.45	
3	21	f	RRMS	4	12	E-pPML	22	18		corticosteroids	6	2.5	2	2.5	2.5	90	90	90	80	1.74	1.70	1.40	2.51	2.13	1.73	0.70	0.80	0.81	0.80	0.61	0.55
4	28	f	RRMS	6	19	E-pPML	49	52		dimethyl fumarate	4	3.5	2	2	3.5	70	100	60	70	0.88	1.63	1.58	1.08	2.65	2.36	0.81	0.61	0.67	1.78	0.40	0.70
5	36	m	RRMS	8	19	E-pPML	48	0		interferon beta	1	1.5	1.5	1.5	1.5	100	100	100	100	1.02	1.35	2.13	1.55	1.56	1.60	0.66	0.87	1.33	1.15	0.57	1.65
6	40	m	RRMS	6	23	L-pPML	48	5		fingolimod	4	4	2	3	3	70	80	60	70	1.27	1.76		1.52	2.15		0.84	0.82		0.72	1.06	
7	43	m	RRMS	9	26	L-pPML JCV+	51	444	54	no	8	5.5	3.5	4	4	60	80	70	60	1.00	1.21		0.90	1.77		1.12	0.68		0.27	0.35	
8	38	f	RRMS	16	27	L-pPML	49	27		interferon beta	7	4	2.5	3	3	80	90	60	70	1.08	1.67	1.21	1.06	1.82	1.02	1.02	0.92	1.18	0.91	0.51	0.48
9	35	f	RRMS	11	29	L-pPML JCV+	62	1390	27	iv. immunoglobulin	4	7.5	1	6	7	50	100	70	50	1.05			0.87			1.21			0.28		
10	47	f	RRMS		38	L-pPML	72	202		no	3	4	2.5	3.5	2	80	90	70	80	1.44	1.62		1.68	3.23		0.85	0.50		0.14	0.42	
11	50	f	RRMS	15	38	L-pPML	40	6000		no	6	5	2	2	4	80	100	70	80	1.42	1.27		1.82	2.84		0.78	0.45		0.12	0.45	
12	38	f	RRMS	5	45	L-pPML	26	37		interferon beta	9	6.5	2	2	6	80	100	70	60	1.29	1.55		1.97	2.61		0.65	0.59		0.52	0.46	
13	35	f	RRMS	9	51	L-pPML JCV+	30	7400	240	no	9	7	1.5	3.5	7	60	90	90	50	0.92	1.88	1.91	1.09	2.59	2.45	0.85	0.73	0.78	0.22	0.75	0.97
14	41	f	RRMS	4	56	L-pPML JCV+	27	42	27	corticosteroids	6	5	1.5	3.5	5	60	90	90	60	1.29	1.39		1.49	1.35		0.86	1.04		0.44	0.41	
15	37	m	RRMS	12	67	L-pPML	34	237		dimethyl fumarate	5	6.5	2	5	6.5	50	90	40	50	1.24	1.49		1.78	2.30		0.70	0.65		0.97	0.42	

MRS=magnetic resonance spectroscopy
MS= multiple sclerosis
RRMS= relapsing-remitting MS
PML= progressive multifocal leukoencephalopathy
E-pPML= early post-PML (<= 19 months)
L-pPML= late post-PML (>= 23 months)
DMT= disease modifying therapy
EDSS=expanded disability status scale
KPS= Karnofsky performance scale
NAWM= normal appearing white matter
NAA=N-acetyl aspartate
Cr=Creatine
Cho=Choline
Lip=lipid

[A]: PML lesion extension score (maximum 9), assessed on axial FLAIR images at time of MRS

PML lesion affecting
1= single gyrus
2= two gyri
3= single lobe
4= multiple lobes
5= one hemisphere
6= multiple lobes in both hemispheres
7= one hemisphere and one contralateral lobe
8= one hemisphere and multiple contralateal lobes
9= both hemispheres
